# Supplementary material for: Association of Circulating microRNAs with Coronary Artery Disease and Usefulness for Reclassification of Healthy Individuals: The REGICOR Study
Source: J Clin Med. 2020 May 9;9(5):1402. doi: 10.3390/jcm9051402 (PMC7290581; doi:10.3390/jcm9051402)
Supplement: Supplementary file 1 [file jcm-09-01402-s001.pdf]

Supplementary Materials

Supplementary figures

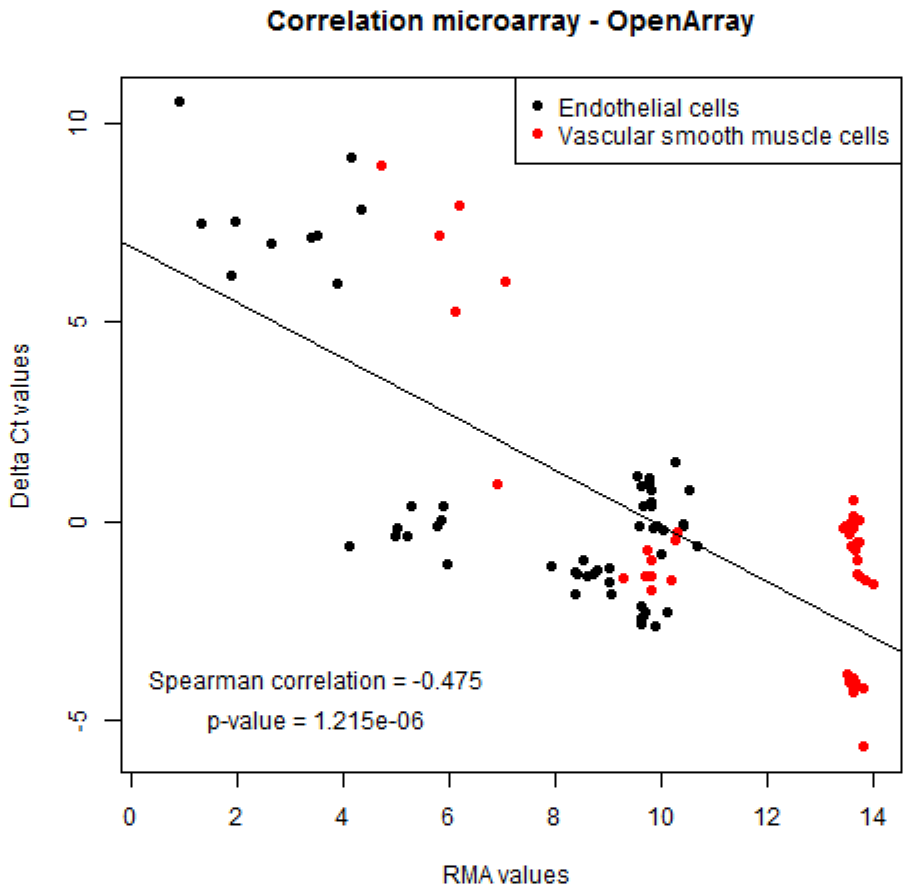

Figure S1. Correlation of RMA values from the microarray and delta Ct values from the Open array, obtained from miRNA expression in human endothelial and smooth muscle cells. Correlation was performed with all miRNAs measured in the custom OpenArray plates: 21 identified in the low density lipoprotein cell treatment study, 22 previously reported to be associated with coronary artery disease, 6 used as housekeeping miRNAs in previous studies, and 2 spike-ins (Table S1). RMA: robust multichip average expression measure.

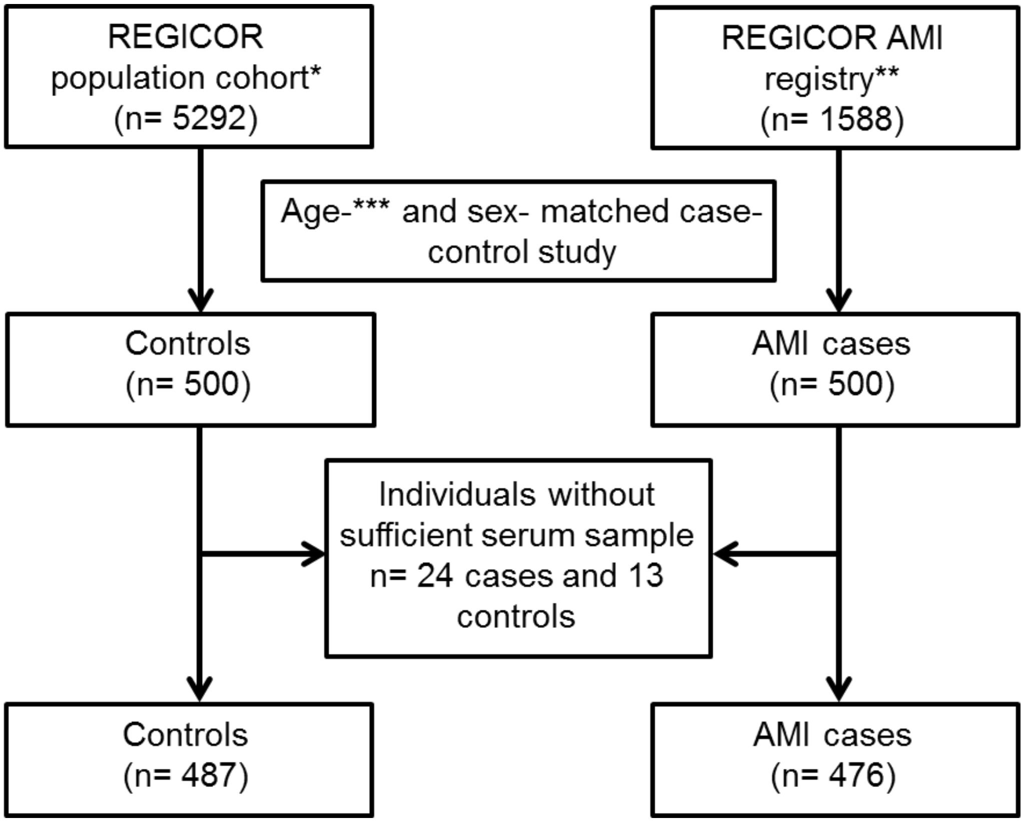

Figure S2. Flowchart of participants in the case-control study**.*** Without prior CVD, aged between 25-74 years, and without CVD events during the follow-up. ** Admitted at the area reference hospital (Trueta Hospital, Girona) during 2001-2006, with all the necessary variables, and aged 25-74 years. *** Matched by age ±1 year. AMI: acute myocardial infarction; REGICOR: Registre Gironí del Cor.

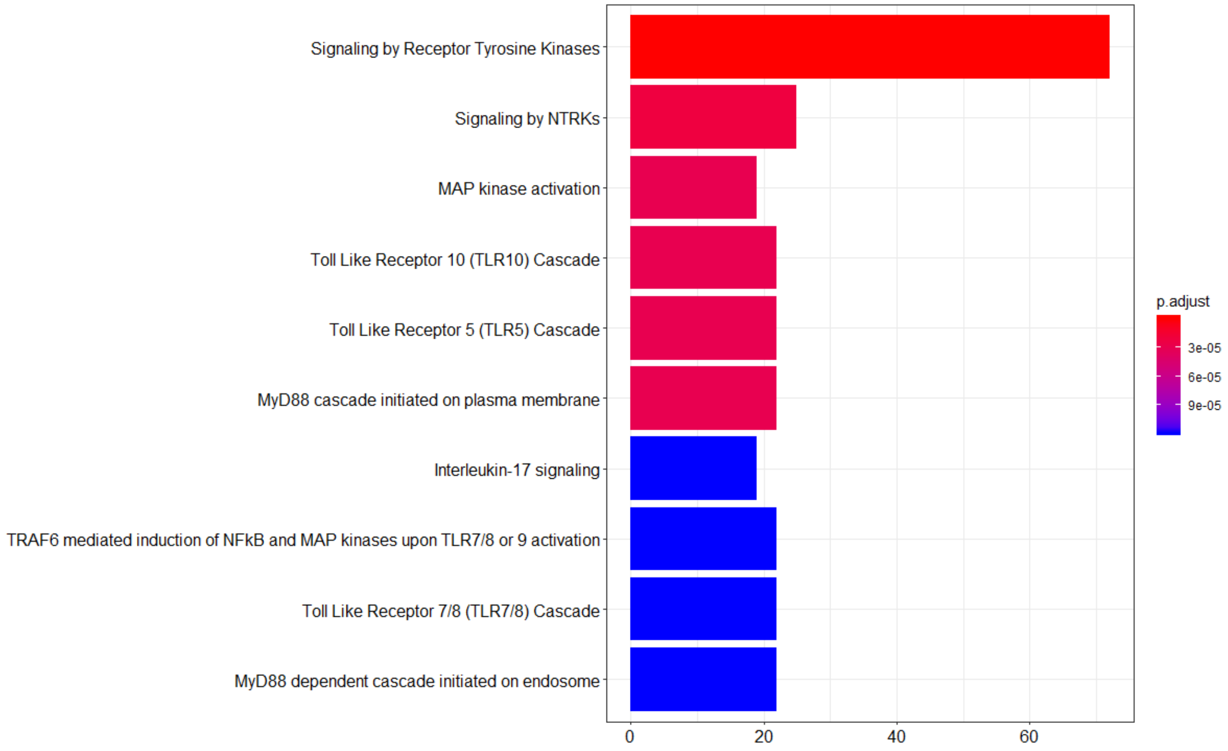

Figure S3. Top 10 most significant pathways associated with the miRNAs differentially expressed in the case-control study. mRNA targets of the 12 differentially expressed miRNA in the case-control study were obtained from TargetScan. Targets with high binding efficacy and conservation were selected using TargetScan scores, and used to analyze the associated pathways in Reactome with the hypergeometric test. The full list with the 53 associated pathways are shown in Supplemental Table 8.

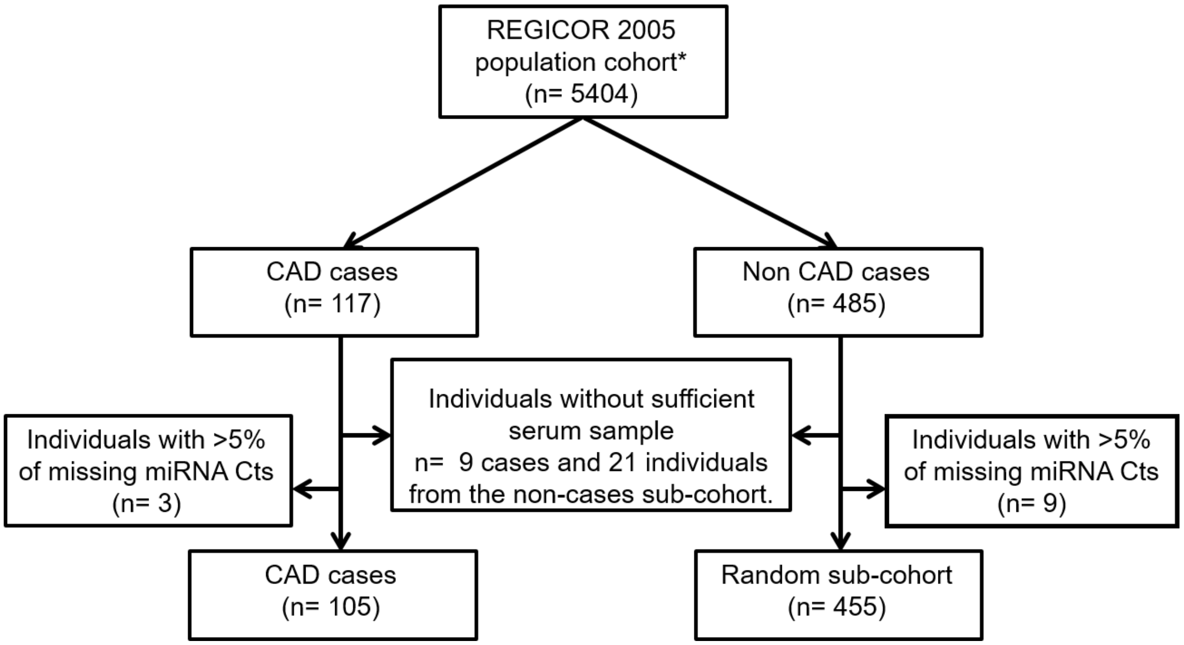

Figure S4. Flowchart of participants in the case-cohort study.* Without prior CVD, aged between 25-74 years, and with available follow-up. CAD: coronary artery disease; Ct: cycle at which the real-time qPCR miRNA sample reaction exceeds the fluorescent intensity above background levels; REGICOR: Registre Gironí del Cor.

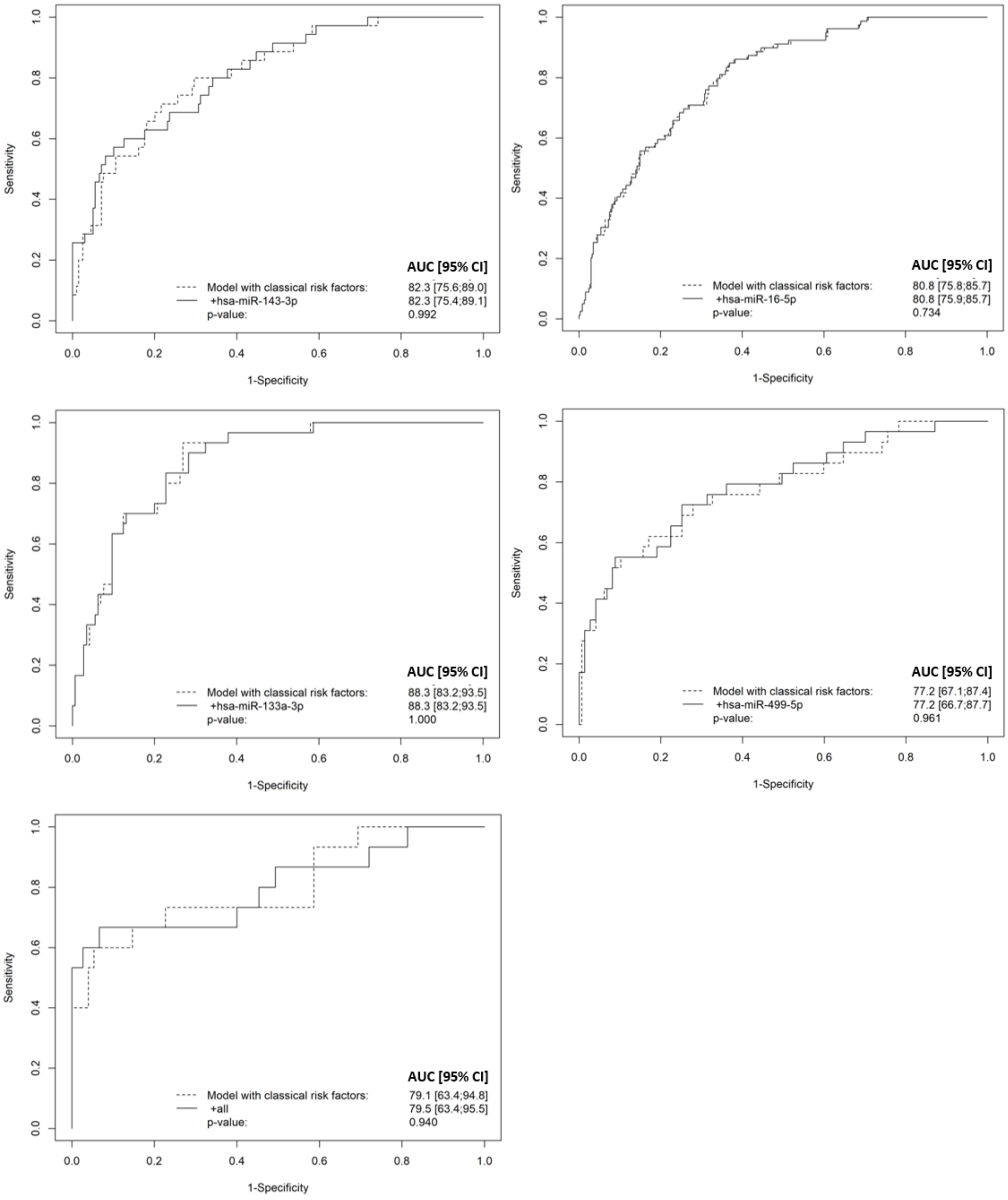

.

Figure S5. Receiver operating characteristic curves and areas under the curve for the Framingham-REGICOR CAD risk function with classical risk factors or classical risk factors plus the selected miRNAs. AUC: area under the receiver operating characteristic curve; CI: confidence interval.

Supplementary tables

Table S1. List of miRNAs included in the custom OpenArray plates and analyzed in the case-control and case-cohort studies.

Identified from the LDL cell treatment study: miRNA expression following a linear or quadratic trend by increasing LDL oxidation treatment		hsa-miR-122-5p<br>hsa-miR-125a-3p<br>hsa-miR-138-1-3p<br>hsa-miR-193b*<br>hsa-miR-197-3p<br>hsa-miR-1229-5p<br>hsa-miR-3064-3p<br>hsa-miR-3151-5p<br>hsa-miR-3176<br>hsa-miR-3656<br>hsa-miR-4632<br>hsa-miR-4634<br>hsa-miR-4669<br>hsa-miR-4676-5p<br>hsa-miR-4732-5p<br>hsa-miR-6728-5p<br>hsa-miR-6795-5p<br>hsa-miR-6831-5p<br>hsa-miR-7107-5p<br>hsa-miR-8085
Identified from the literature	Used as housekeeping miRNAs in previous studies	hsa-miR-16-5p<br>hsa-miR-22-3p<br>hsa-miR-24-3p<br>hsa-miR-31-5p<br>hsa-miR-191-5p<br>hsa-miR-222-3p
	Associated with CAD in previous studies	hsa-miR-1*<br>hsa-miR-21*<br>hsa-miR-125b-5p<br>hsa-miR-133a*<br>hsa-miR-133b<br>hsa-miR-134*<br>hsa-miR-143*<br>hsa-miR-145*<br>hsa-miR-146a*<br>hsa-miR-186*<br>hsa-miR-208b*

	hsa-miR-499*
Spike-ins	ath-miR-159a<br>cel-miR-2-3p

*miRNAs for which both the 3p and the 5p were analyzed.

Table S2. Articles reviewed to define athero-protective and athero-prone gene sets in ECs and VSMCs.

Main authors	Reference
Endothelial cells	
Gimbrone M.A. Jr, García-Cardeña G.	*Circ Res*. **2016**, *118*, 620-636.
Dai G., Kaazempur-Mofrad M.R., Natarajan S., Zhang Y., et al.	*Proc Natl Acad Sci U S A*. **2004**, *101*, 14871-14876
Basatemur G.L., Jørgensen H.F., Clarke M.C.H., et al.	*Nat Rev Cardiol*. **2019**, *16*, 727-744.
**Vascular smooth muscle cells**	
Basatemur G.L., Jørgensen H.F., Clarke M.C.H., et al.	*Nat Rev Cardiol*. **2019**, *16*, 727-744.
Chettimada S., Joshi S.R., Dhagia V., et al.	*Am J Physiol Heart Circ Physiol*. **2016**, *311*, H904-H912.
Beamish J.A., He P., Kottke-Marchant K., et al.	*Tissue Eng Part B Rev*. **2010**, *16*, 467-491.
Chang S., Song S., Lee J., et al.	*PLoS One*. **2014**, *9*, e88089.
Wanjare M., Kuo F., Gerecht S.	*Cardiovasc Res*. **2013**, *97*, 321-330.
Xu F., Ahmed A.S., Kang X., et al.	*Arterioscler Thromb Vasc Biol*. **2015**, *35*, 2145-2152.
Wang X., Hu G., Gao X., et al.	*Arterioscler Thromb Vasc Biol*. **2012**, *32*, 2662-2669.
Cattaruzza M., Nogoy N., Wojtowicz A., et al.	*FASEB J*. **2012**, *26*, 4864-4875.

Table S3. Characteristics of the individuals whose samples were used in the low density lipoprotein cell treatment study.

	**Individuals from the COLMAH Collection n=3**
Age (years)	73.7 ± 8.1
Gender (% female)	1 (33%)
Smoking (%)	2 (66%)
Total cholesterol (mmol/L)	5.69 ± 0.45
Triglycerides (mmol/L)	1.84 ± 0.13
Systolic blood pressure (mm Hg)	157 ± 5.8
Diastolic blood pressure (mm Hg)	103 ± 11.6
Glucose (mmol/L)	7.83 ± 2.43
Creatinine (mmol/L)	0.09 ± 0.06

Data are shown as mean ± standard deviation except for gender and smoking, for which the number of individuals and the percentage is presented.

Table S4. Characteristics of the case-control study participants included in the analysis.

	Controls N=487	AMI cases N=476	p-value
Age (years)	60.1 ± 9.99	60.5 ± 9.86	0.567
Gender (% female)	387 (76%)	376 (79%)	0.919
Smoking (%)	123 (26%)	264 (56%)	<0.001
Previous history dyslipidemia (%)	169 (35%)	250 (59%)	<0.001
Previous history hypertension (%)	190 (40%)	285 (64%)	<0.001
Previous history diabetes (%)	70 (15%)	147 (33%)	<0.001
Body mass index (kg/m^2)	27.6 ± 3.82	28.0 ± 4.28	0.134

Data are shown as number of individuals and percentage, except for age and body mass index, for which mean ± standard deviation is presented.
p-values were not adjusted for multiple comparisons.

Table S5. Baseline characteristics of the case-cohort study participants.

	Individuals from the random cohort subsample n=455	Individuals that had a CAD within 10 years n=105	p-value
Age (years)	54.3 ± 11.0	61.1 ± 9.56	<0.001
Gender (% female)	240 (53%)	34 (32%)	0.001
Smoking (%)	96 (21%)	38 (37%)	0.002
Total cholesterol (mmol/L)	5.46 ± 1.09	5.90 ± 1.10	<0.001
LDL cholesterol (mmol/L)	3.52 ± 0.94	3.85 ± 0.99	0.002
HDL cholesterol (mmol/L)	1.36 ± 0.35	1.2 ± 0.37	<0.001
Triglycerides (mmol/L)	1.21 ± 0.64	1.86 ± 1.52	<0.001
Systolic blood pressure (mm Hg)	126 ± 18.9	138 ± 19.7	<0.001
Diastolic blood pressure (mm Hg)	78.6 ± 9.8	82.4 ± 11.0	<0.001
Glucose (mmol/L)	5.44 ± 1.12	6.49 ± 2.59	<0.001
Creatinine (mmol/L)	0.08 ± 0.02	0.08 ± 0.02	0.485
Body mass index (kg/m^2)	27.7 ± 5.0	28.5 ± 4.6	0.302

Data are shown as mean ± standard deviation except for gender and smoking, for which the number of individuals and the percentage are presented. p-values were not adjusted for multiple comparisons.

Table S6. miRNAs differentially expressed in human endothelial cells and vascular smooth muscle cells treated with oxidized low density lipoproteins (LDL) compared with native LDLs (nLDL), and following significant linear/quadratic trend across nLDL, moderately oxidized LDL (moxLDL) and highly oxidized LDL (hoxLDL) in at least one cell type.

miRNA	Endothelial cells log2(fold change)	Fold change	p-value	Vascular smooth muscle cells log2(fold change)	Fold change	p-value
hsa-miR-122-5p	NA	NA	NA	1.380 (1)	**2.603**	**<0.001**
hsa-miR-125a-3p	-1.081 (2)	**0.473**	**0.031**	0.083 (1)	1.060	0.762
hsa-miR-138-1-3p	NA	NA	NA	-0.745 (1)	**0.597**	**0.007**
hsa-miR-193b-3p	-0.781 (2)	**0.582**	**0.017**	-0.112 (2)	0.925	0.507
hsa-miR-193b-5p	-0.872 (2)	**0.546**	**0.004**	0.100 (1)	1.072	0.589
hsa-miR-197-3p	-1.975 (2)	**0.254**	**0.001**	-0.104 (1)	0.930	0.567
hsa-miR-1229-5p	-0.627 (1)	**0.648**	**0.002**	-0.425 (1)	0.745	0.285
hsa-miR-3064-3p	-0.753 (2)	**0.593**	**0.036**	-0.137 (1)	0.909	0.440
hsa-miR-3151-5p	-0.505 (2)	**0.705**	**0.033**	0.593 (1)	**1.509**	**0.006***
hsa-miR-3176	NA	NA	NA	-0.598 (1)	**0.660**	**0.004**
hsa-miR-3656	-0.239 (2)	0.847	0.627	0.669 (1)	**1.590**	**0.006**
hsa-miR-4632	NA	NA	NA	0.659 (1)	**1.579**	**0.006**
hsa-miR-4634	-0.986 (1)	0.505	0.094	0.693 (1)	**1.616**	**0.005**
hsa-miR-4669	-0.834 (1)	**0.561**	**0.037**	-0.596 (1)	**0.662**	**0.012**
hsa-miR-4676-5p	NA	NA	NA	0.677 (1)	**1.599**	**0.002**
hsa-miR-4732-5p	-0.700 (2)	**0.615**	**0.049**	0.232 (2)	1.174	0.312
hsa-miR-6728-5p	-0.745 (2)	0.597	0.125	-1.015 (1)	**0.495**	**0.002**
hsa-miR-6795-5p	-0.628 (2)	**0.647**	**0.014**	-0.240 (1)	0.847	0.556
hsa-miR-6831-5p	-0.759 (2)	**0.591**	**0.032**	-0.614 (1)	**0.653**	**0.022**
hsa-miR-7107-5p	0.640 (2)	**1.559**	**0.020**	-0.375 (1)	**0.771**	**0.046**
hsa-miR-8085	-0.775 (1)	**0.584**	**<0.001***	0.375 (1)	1.296	0.155

hoxLDL *vs.*, nLDL, (2) moxLDL *vs.*, Nldl, * Expression change was significant in comparisons (1) and (2), p-values were not adjusted for multiple comparisons.

Table S7. Adjusted p-values of linear/quadratic trend across native low density lipoproteins (nLDL), moderately oxidized LDL (moxLDL) and highly oxidized LDL (hoxLDL). The miRNAs were selected when significant or marginally significant in at least one cell type.

miRNA	Endothelial cells Linear trend	Quadratic trend	Vascular smooth muscle cells Linear trend	Quadratic trend
hsa-miR-122-5p	NA	NA	**0.024**	0.430

hsa-miR-125a-3p	0.818	0.051	NA	NA
hsa-miR-138-1-3p	NA	NA	**0.024**	0.392
hsa-miR-193b-3p	0.937	**<0.001**	NA	NA
hsa-miR-193b-5p	0.937	0.051	NA	NA
hsa-miR-197-3p	0.987	**0.047**	NA	NA
hsa-miR-1229-5p	0.321	**0.014**	NA	NA
hsa-miR-3064-3p	0.927	**0.014**	NA	NA
hsa-miR-3151-5p	NA	NA	0.052	0.540
hsa-miR-3176	NA	NA	**0.031**	0.597
hsa-miR-3656	NA	NA	0.052	0.469
hsa-miR-4632	NA	NA	**0.024**	0.956
hsa-miR-4634	NA	NA	0.052	0.336
hsa-miR-4669	0.372	0.763	0.052	0.767
hsa-miR-4676-5p	NA	NA	0.024	0.767
hsa-miR-4732-5p	0.617	**0.007**	NA	NA
hsa-miR-6728-5p	NA	NA	**0.031**	0.469
hsa-miR-6795-5p	0.937	0.051	NA	NA
hsa-miR-6831-5p	0.372	0.763	0.052	0.767
hsa-miR-7107-5p	0.818	0.051	NA	NA
hsa-miR-8085	**0.046**	0.172	NA	NA

Missing observations correspond to miRNAs without data for the specific cell type or that had a Fold Change <1.5., p-values were adjusted for multiple comparisons.

Table S8. miRNA expression in the case-control study: fold change of cases *vs.*, controls.

miRNA	N	log2(fold change)	Fold change	adjusted p-value
hsa-miR-16-5p	890	3.426	**10.745**	**<0.001**
hsa-miR-21-5p	751	2.743	**6.696**	**<0.001**
hsa-miR-22-3p	859	1.114	**2.164**	**<0.001**
hsa-miR-24-3p	947	0.014	1.010	0.228
hsa-miR-122-5p	591	2.276	**4.845**	**<0.001**
hsa-miR-125b-5p	924	0.431	**1.348**	**<0.001**
hsa-miR-133a-3p	455	3.300	**9.851**	**<0.001**
hsa-miR-143-3p	624	2.397	**5.268**	**<0.001**
hsa-miR-145-5p	671	1.284	**2.435**	**<0.001**
hsa-miR-146a-5p	909	0.428	**1.345**	**0.006**
hsa-miR-186-5p	829	1.678	**3.200**	**<0.001**
hsa-miR-191-5p	871	0.217	1.162	0.081
hsa-miR-222-3p	739	3.170	**8.999**	**<0.001**
hsa-miR-499a-5p	595	6.885	**118.186**	**<0.001**

p-values were adjusted for multiple comparisons.

Table S9. Pathways associated with the targets with higher binding efficacy and conservation for the miRNA associated to myocardial infarction discovered in the case-control study.

Pathway	Number of targets*	Adjusted p-value
Signaling by Receptor Tyrosine Kinases	72	<0.001

Signaling by NTRKs	25	<0.001
MAP kinase activation	19	<0.001
Toll Like Receptor 10 (TLR10) Cascade	22	<0.001
Toll Like Receptor 5 (TLR5) Cascade	22	<0.001
MyD88 cascade initiated on plasma membrane	22	<0.001
Interleukin-17 signaling	19	<0.001
TRAF6 mediated induction of NFkB and MAP kinases upon TLR7/8 or 9 activation	22	<0.001
Toll Like Receptor 7/8 (TLR7/8) Cascade	22	<0.001
MyD88 dependent cascade initiated on endosome	22	<0.001
MyD88:MAL(TIRAP) cascade initiated on plasma membrane	22	<0.001
Toll Like Receptor TLR6:TLR2 Cascade	22	<0.001
Toll Like Receptor 9 (TLR9) Cascade	22	<0.001
Toll Like Receptor TLR1:TLR2 Cascade	22	<0.001
Toll Like Receptor 2 (TLR2) Cascade	22	<0.001
Signaling by NTRK1 (TRKA)	19	<0.001
Activation of BH3-only proteins	11	<0.001
Negative regulation of the PI3K/AKT network	22	<0.001
Toll Like Receptor 3 (TLR3) Cascade	21	<0.001
Signaling by FGFR1	14	<0.001
MyD88-independent TLR4 cascade	21	<0.001
TRIF(TICAM1)-mediated TLR4 signaling	21	<0.001
Intrinsic Pathway for Apoptosis	13	<0.001
L1CAM interactions	23	<0.001
Transcriptional Regulation by TP53	50	<0.001
Diseases of signal transduction	51	<0.001
Negative regulation of FGFR2 signaling	11	<0.001
Signaling by FGFR3	12	<0.001
Negative regulation of FGFR3 signaling	10	<0.001
Toll Like Receptor 4 (TLR4) Cascade	24	<0.001
MAPK family signaling cascades	42	<0.001
Neurotoxicity of clostridium toxins	6	<0.001
PI5P, PP2A and IER3 Regulate PI3K/AKT Signaling	20	0.001
MAPK targets/ Nuclear events mediated by MAP kinases	10	0.002
MAPK1/MAPK3 signaling	37	0.002
RAF/MAP kinase cascade	36	0.002
Negative regulation of FGFR1 signaling	10	0.003
Negative regulation of MAPK pathway	11	0.003
ERK/MAPK targets	8	0.003
Signaling by FGFR4	11	0.004
Signaling by Interleukins	56	0.005
Chk1/Chk2(Cds1) mediated inactivation of Cyclin B:Cdk1 complex	6	0.005
Clathrin-mediated endocytosis	23	0.007
Negative regulation of FGFR4 signaling	9	0.008
Nuclear Events (kinase and transcription factor activation	8	0.008
Recycling pathway of L1	11	0.009

Other interleukin signaling	8	0.010
PIP3 activates AKT signaling	35	0.010
Toll-like Receptor Cascades	24	0.011
Activation of BAD and translocation to mitochondria	6	0.011
Insulin receptor signalling cascade	12	0.011
Signaling by PTK6	12	0.011
Signaling by Non-Receptor Tyrosine Kinases	12	0.011

Number of targets present in each pathway from the list of targets with higher binding efficacy and conservation (n=1431). p-values were adjusted for multiple comparisons.

Table S10. miRNA expression effect (hazard ratio and 95% confidence interval (CI)) adjusted for age and sex in the case-cohort study.

miRNA	N	Hazard ratio	95% CI
hsa-miR-16-5p	475	1.026	[0.928, 1.134]
hsa-miR-21-5p	352	0.896	[0.675, 1.189]
hsa-miR-22-3p	423	1.049	[0.796, 1.382]
hsa-miR-24-3p	544	1.083	[0.842, 1.417]
hsa-miR-122-5p	245	1.081	[0.846, 1.381]
hsa-miR-125b-5p	532	1.004	[0.828, 1.223]
hsa-miR-133a-3p	184	0.952	[0.743, 1.221]
hsa-miR-143-3p	248	**0.562**	**[0.384, 0.824]**
hsa-miR-145-5p	312	0.920	[0.681, 1.243]
hsa-miR-146a-5p	482	1.193	[0.922, 1.545]
hsa-miR-186-5p	424	0.942	[0.792, 1.120]
hsa-miR-191-5p	448	0.971	[0.756, 1.235]
hsa-miR-222-3p	333	1.075	[0.854, 1.354]
hsa-miR-499a-5p	189	0.934	[0.750, 1.164]

Table S11. Athero-protective and athero-prone hsa-miR-143-3p targets identified in ECs and SMCs.

**EC athero-protective targets**	**MAPK7: Mitogen-Activated Protein Kinase 7**<br>**PODXL: Podocalyxin Like**<br>**PPAP2B: Phospholipid phosphatase 3**<br>**SENP2: SUMO Specific Peptidase 2**
EC athero-prone targets	CTGF: *connective tissue growth factor*<br>SERPINE1: Serpin Family E Member 1<br>VTI1B: Vesicle Transport Through Interaction With T-SNAREs<br>THBS1: Thrombospondin 1<br>NRG1: Neuregulin 1
VSMC athero-protective targets	CACNA1C: Calcium Voltage-Gated Channel Subunit Alpha1 C
VSMC athero-prone targets	COL1A1: Collagen Type I Alpha 1 Chain<br>PTGS2: Prostaglandin-Endoperoxide Synthase 2
